# Differential Replication for Credit Scoring in Regulated Environments

**DOI:** 10.3390/e23040407

**Published:** 2021-03-30

**Authors:** Irene Unceta, Jordi Nin, Oriol Pujol

**Affiliations:** 1 BBVA Data & Analytics, 28050 Madrid, Spain; irene.unceta@esade.edu; 2 ESADE, Universitat Ramon Llull, 08172 Sant Cugat del Vallès, Spain; 3 Department of Mathematics and Computer Science, Universitat de Barcelona, 08007 Barcelona, Spain; oriol_pujol@ub.edu

**Keywords:** differential replication, environmental adaptation, copying, credit scoring

## Abstract

Differential replication is a method to adapt existing machine learning solutions to the demands of highly regulated environments by reusing knowledge from one generation to the next. Copying is a technique that allows differential replication by projecting a given classifier onto a new hypothesis space, in circumstances where access to both the original solution and its training data is limited. The resulting model replicates the original decision behavior while displaying new features and characteristics. In this paper, we apply this approach to a use case in the context of credit scoring. We use a private residential mortgage default dataset. We show that differential replication through copying can be exploited to adapt a given solution to the changing demands of a constrained environment such as that of the financial market. In particular, we show how copying can be used to replicate the decision behavior not only of a model, but also of a full pipeline. As a result, we can ensure the decomposability of the attributes used to provide explanations for credit scoring models and reduce the time-to-market delivery of these solutions.

## 1. Introduction

In most real-life company deployment scenarios, machine learning models are only a small part of the larger structure entailed by a machine learning system [1]. This structure accounts for all the elements that interact with a model throughout its lifespan. A model’s context includes everything from the data and its sources to the deployment infrastructure, the governance protocol or the more general regulatory framework. This context includes dimensions which are both internal and external to the organization and which are mostly out of our control [2,3]. More worryingly, these are all dimensions that refer to constraints prone to change in time [4].

Privacy restrictions may limit the usability of the data or the models themselves [5,6,7]. A modification in the regulatory framework may require models to be self-explanatory [8,9,10] or fair with respect to sensitive data attributes [11,12,13]. A company’s production environment may change, requiring the whole production pipeline to be updated in order to deliver certain solutions to the market [3]. Such changes in a machine learning model’s environment introduce new constraints that may render the previous set of feasible solutions obsolete and require the model to adapt these circumstances.

This problem has been defined as environmental adaptation [14] and refers to situations where the learning task remains the same, but the environmental conditions change. Contrary to what one may think, changes in the environmental conditions of a model happen with a relatively high frequency and may arise from a change in the technological infrastructure, the business needs, the existing legislation, etc. The new environmental conditions can be formally defined as a set of new constraints. As a result of these constraints, the existing solution is left out of the new feasible set. The adaptation problem consists of finding a new solution that solves the given task, while satisfying the new constraints. In this context, *differential replication* has been proposed as a technique to reuse the knowledge acquired by the deployed models to train more suitable future generations by projecting the existing solutions onto the new feasible sets. Depending on the considered level of knowledge, there exist different inheritance mechanisms that implement differential replication in practice. In this paper, we are interested in using *inheritance by copying* [15]. In particular, we discuss differential replication through copying in the context of non-client credit risk scoring.

A growing trend in credit risk scoring is to increase model sophistication, in the hope that models can learn ever more complex problems with a high degree of accuracy. This has led to the proliferation of so-called “black-box” systems, which fail to provide a comprehensible account of how they reach their conclusions. Even while such systems may yield good performance, they generally do so at the cost of simplicity and understandability. This is a situation that stands in contrast to the growing demand for transparency and accountability of automated processing models [8,9]. More so in the financial industry, a sector that is highly regulated and where loan issuers are required by law to explain the credit models used to approve or to decline loan applications. In this paper, we exploit differential replication through copying to solve issues related to model interpretability under different assumptions. Namely, that either the input variables or the model itself are obfuscated in order to optimize accuracy.

Our primary technical contribution is a twofold solution to the problem of interpreting credit risk scoring models in this circumstances. On the one hand, we de-obfuscate preprocessed input variables by building copies directly on the raw data attributes. We do so by copying a whole predictive pipeline. On the other hand, we show how the preprocessing step can be avoided by training a more complex model and then substituting it with an interpretable copy that retains a good overall performance. As a result, we deliver credit scoring models that are compliant with the regulatory demands for understandability.

The remainder of this paper is organized as follows. First, Section 2 presents a literature survey of related work. In Section 3, we derive the theoretical framework for copying and describe how copies are built in practice. In Section 4, we discuss differential replication in the context of non-client credit scoring and present a real-life use case. Our experimental results are presented in Section 5, where we describe the applicability of copies in two well-established scenarios. Finally, the paper ends with a summary of our conclusions and points out directions for future discussion and improvement.

## 2. Related Work

Failure to keep with loan repayment, otherwise known as credit default, has significant cost implications for financial institutions. Credit scoring refers to the area of knowledge concerned with developing empirical models to support decision making in the retail credit business. Decisions output by these models have a substantial impact on people’s life, as they regulate the pricing and availability of mortgages and therefore affects consumers’ disposable income. Therefore, the use of automatic decision-making systems for credit scoring by banks has been traditionally subject to great scrutiny by national and international financial regulators [16,17,18,19].

These regulators require, among other specifications, that internal coefficients and variable importance of credit scoring models be accessible [9] and in line with human domain knowledge [20,21]. This largely limits the type of models that can be used in practice in this type of setting. When a financial institution trains a credit risk model, prediction accuracy is of paramount importance, i.e., companies aim to maximize revenue through model accuracy. Yet, interpretability is a legal requirement [22]. This constitutes a conundrum for most companies [21]: it is generally accepted that there exists a complex trade-off between accuracy and interpretability, whereby maximization of one comes at the expense of the other. Companies are therefore often faced with the need of having to give up one for the sake of the other.

With the aim of overcoming these issues, several works have advocated for the use of models that ensure interpretability by design while being able to reach a reasonable level of complexity [23]. In addition, noniterative supervised learning models [24] have also been shown to provide a fast alternative to both classification and regression models with increased accuracy. Yet, logistic regression remains the most widely established technique for ensuring interpretability in the credit risk modeling context [25,26,27,28]. Models based on logistic regression perform reasonably well on default prediction settings, while at the same time offering the additional advantage of a relative ease of interpretation. Not in vain do institutions such as FICO train their credit models using this architecture, concretely mentioning interpretability as one of the main motivations for it [29].

A main drawback of logistic regression models, however, is that they are linear. They therefore fail to account for nonlinearities in the data. A failure that usually results in the loss of accuracy. To overcome this limitation, nonlinear effects are usually modeled during the preprocessing step. During this step, which is prior to model training, domain knowledge by experts is exploited to obtain a set of highly predictive, artificially generated attributes that ensure a good predictive performance for the logistic regression model. This practice is, however, against the idea of *intelligibility* as described in [30]. Nonlinear attributes generally lack a meaning by themselves; more so in cases where they are generated as combinations of the raw variables. Therefore, preprocessing often results in *non-decomposable* [31] machine learning pipelines that do not comply with the existing regulation. This problem is different from that of ensuring the use of an interpretable model. Previous works devoted to explaining how machine learning models work generate local explanations around a set of predictions [32,33]. To increase model transparency by offering interpretability at a global scale, those local explanations are integrated using a framework for interpreting predictions known as SHapley Additive exPlanations (SHAP) [34] based on aggregations of Shapley values [35]. An alternative to SHAP is the permutation feature importance measure [36]. This measure evaluates the importance of a feature by calculating the increase in the model’s prediction error after permuting each attribute. An attribute is considered important when shuffling its values increases the model error. Even while such explanations may faithfully represent the functioning of a model locally, note that they are based on non-interpretable data attributes and hence often fail to meet regulatory requirements.

To circumvent this issue, one might use more complex models, such as deep artificial neural networks [37], that capture the nonlinearities in the data [38]. More so, because in credit scoring problems, models needs to deal with imbalanced data sets [39,40] as the number of defaulting loans in a financial institution portfolio is much lower than non-defaulted [41] or issued [42] ones. However, this approach results in machine learning solutions whose internals cannot be inspected. The problem of balancing accuracy and interpretability in credit risk scoring therefore remains unsolved. In this article, we propose differential replication through copying as a way around this compromise.

## 3. Adapting Models to the Demands of Their Environment

Differential replication is a particular case of model adaptation to changes in its environment. The use of copies for this purpose is further particularization of this problem to a concrete use case. In what follows, we introduce the notion of adaptation and describe differential replication as a technique to adapt models to changing conditions. We begin by explaining the differences between this approach and other techniques existing in the literature.

### 3.1. Environmental Adaptation and Differential Replication

The idea of adaptation is present in different areas of the machine learning literature. In its simplest form, adaptation refers to how data practitioners put theoretical proposals to practice in their everyday life [43,44,45]. Other forms of adaptation are related to situations where the underlying data distribution changes resulting in a concept drift [46]. In general, this happens in the presence of data streams [47]. Traditional batch learners are incapable of adapting to such drifts. Instead, online learning algorithms can iteratively update their knowledge according to changes in the data [48].

A discipline that studies changes in data distribution is *transfer learning*. In particular, it focuses on cases where learning a given task can be improved through the transfer of knowledge acquired when learning a related task [49,50,51]. In certain transfer learning problems, the change of task is accompanied by a change in the domain, so that data labeled in a single [52] or multiple [53] source domains are leveraged to learn a classifier on unseen data in another domain. This is the case of domain adaptation [52,53], which deals with learning knowledge representations for one domain such that they can be transferred to another related target domain.

In all these cases, the defined hypothesis space remains valid; this is, it still encompasses a set of acceptable solutions for the considered problem. Yet, the original solution needs to be adapted to the new domain or task. There are situations, however, where the existing solution is rendered obsolete or where the new feasible set does not overlap with the defined hypothesis space. These are situations where it is not the data distributions or the problem domain that change, but a model’s environment itself. As defined in [14], a machine learning model’s environment comprises all the elements that interact with the model throughout its lifespan, including the data and their different sources, the deployment infrastructure, the governance protocol, or the regulatory framework. This environment can be mathematically formalized as a set of constraints on the hypothesis space. Changes to this environment, therefore involves the appearance of new such constraints. Say that one of the original input attributes is no longer available, that a deployed black-box solution is required to be interpretable or that updated software licenses require moving our current machine learning system to a new production environment. These changes generally require the definition of a new model in a different hypothesis space. It is these situations that the *environmental adaptation* is concerned with [14].

There exist different ways in which to tackle the environmental adaptation problem. In this paper, we are interested in *differential replication*, which enables model survival in changing environments by building on the knowledge acquired by previously trained generations of models.

### 3.2. Methodologies for Differential Replication

The idea of differential replication is based on the biological process of DNA replication, whereby replicas of the same genome are subsequently modified by introducing small changes that ensure a better adaptation to the environment. Following the genetic simile, differential replication refers to the process of changing the genotype (internals of the machine learning model) while retaining the interesting phenotype features (decision boundary) and adding some new ones (introduced by the engineered difference to be boosted or added). When it comes to machine learning systems, this process amounts to creating a new model of a potentially different family (genotype) that retains the same decision behavior (original phenotype) of a given solution, while displaying additional features not present in the original classifier (engineered phenotype traits).

Consider two different model hypothesis spaces—$H_{A}$ and $H_{B}$—and two classifiers, $f_{A}$ and $f_{B}$, belonging to $H_{A}$ and $H_{B}$, respectively. In general, while $f_{A}$ and $f_{B}$ may display the same decision behavior, i.e., classify all points equally, their architecture is not the same. Say, for example, that $f_{A}$ belongs to the family of logistic regression models, while $f_{B}$ is a decision tree. In this case, while both models may converge to the same solution, they are expressed in different ways. The fact that different models can display the same or a close decision behavior is what enables differential replication. The decision function learned by a model can be replicated by another, which may display additional features not present in the original classifier and which can therefore be better suited to meet the instantaneous demands of the environment.

Differential replication can be attained in many different ways. The different techniques differ from each other in the amount of knowledge that is assumed about the initial data and model. In all cases, the different techniques assume some form of inheritance from the existing model that allows the acquired knowledge to be transferred. In the simplest form, inheritance can be attained by sharing the same training data, i.e., by retraining the models [54]. Alternatively, model wrappers can also be used to envelope the existing solutions with an additional learnable layer that enables adaptation [55,56]. Other inheritance methods include data editing [57,58,59] or data enrichment. In particular, mechanisms like distillation [60,61,62,63], label regularization [64,65], or label refinery [66] have been successfully employed.

All these inheritance techniques require some form of access to the source model’s internals and are performed under the assumption of full knowledge of the training data. There exists situations, however, where this assumption does not hold. In this paper, we study one of such situations, where we assume no knowledge about the original model, its internals, or its training data. Under such circumstances, we can ensure differential replication through *copying*. This is the most general form of differential replication and can be performed in absence of any previous knowledge, other than the original model’s prediction outputs over a set of artificially generated data points. In what follows, we provide a brief overview of the copying process. A formal treatment of this process, as well as a display of its applications can be found in [15].

### 3.3. Background on the Copying Process

Given a set of training data points $D={\left\{\left(x_{i},t_{i}\right)\right\}}_{i=1}^{M}$, for *M* the number of samples, we define a classifier as a function $f_{O}:X\to T$ from the sample space X to the label space T. We restrict to classification of real-valued attributes, so that $X=R^{d}$ and $T=Z_{k}$ for *k* the number of classes. We define a copy as a new classifier $f_{C}\left(\theta \right)$, parameterized by θ, such that its decision function mimics $f_{O}$ all over the space. Building a copy, amounts to finding the optimal parameter values $\theta^{*}$, such that $\theta^{*}=\arg\max_{\theta }P\left(\theta |f_{O}\right)$.

However, in the most general case, we cannot assume access to the original training data D. This data may be lost, unknown, or just not accessible. Whatever the reason, because the training data D is unknown we cannot resort to it, nor can we estimate its distribution. To find $\theta^{*}$, we need to explore the sample space by other means to gain information about the specific form of $f_{O}$. Therefore, we introduce synthetic data points $z_{j}∼P_{Z},\;z_{j}\in X$ such that

(1) $$\theta^{*}=\arg\max_{\theta }\int_{z∼P_{Z}}P\left(\theta |f_{O}\left(z\right)\right)dP_{Z}$$

for an arbitrary generating probability distribution $P_{Z}$, from which the synthetic samples are independently drawn. We assume an exponential family form for all probability distributions and rewrite (1) as

(2) $$\begin{array}{cc} \theta^{*} & =\arg\min_{\theta }\left[\int_{z∼P_{Z}}\gamma_{1}\ell_{1}\left(f_{C}\left(z,\theta \right),f_{O}\left(z\right)\right)dP_{Z}+\gamma_{2}\ell_{2}\left(\theta ,\theta^{+}\right)\right], \end{array}$$

for $\ell_{1}$ and $\ell_{2}$ a measure of the disagreement between the two models, and $\theta^{+}$ our prior knowledge of θ.

This optimization problem is always separable and, given enough capacity, zero training error is always achievable without hindering the generalization performance of the copy. In this context, we argue that ad hoc techniques can be used to better exploit these properties when building a copy [15]. In particular, assuming a sufficiently large set of synthetic data points is obtained, we can train the copies with no regard for overfitting. On the contrary, we may want to force the copy to overfit to the synthetic data, to ensure a good fit of the copy decision boundary.

In practice, we cannot generate infinite synthetic samples. Therefore, direct computation of (2) is not possible. Instead, we refer to the regularized empirical risk minimization framework [67] and approximate the expression above as

(3) $$\begin{array}{cc} \left(\theta^{*},Z^{*}\right) & =\arg\min_{\theta ,z_{j}\in Z}\left[\frac{1}{N}\sum_{j=1}^{N}\gamma_{1}\ell_{1}\left(f_{C}\left(z_{j},\theta \right),f_{O}\left(z_{j}\right)\right)+\gamma_{2}\ell_{2}\left(\theta ,\theta^{+}\right)\right] \end{array}$$

where $Z={\left\{z_{j}\right\}}_{j=1}^{N},\;z_{j}∼P_{Z}$. We label this set according to the class prediction outputs of $f_{O}$ and define the synthetic dataset $Z={\left\{\left(z_{j},f_{O}\left(z_{j}\right)\right)\right\}}_{j=1}^{N}$.

The expression above corresponds to a dual optimization problem, where we simultaneously optimize the parameters θ and the set Z. In the simplest approach, we cast this problem into one where we use a single iteration of an alternating projection optimization scheme: the *single-pass copy*.

### 3.4. Approximating the Copying Process with the Simplified Single-Pass Copy

**Figure 1:**
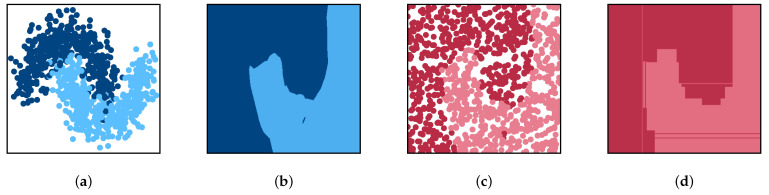
(**a**) Binary classification dataset, (**b**) decision function learned by an artificial neural network, (**c**) synthetic data points sampled uniformly at random from the original feature space and labeled according to the predictions of the neural net, and (**d**) decision function of a decision tree-based copy.

Copying involves the joint optimization of the synthetic data points Z and the parameters θ of the copying model through Equation (3). This joint optimization is a complex process that requires the copy hypothesis space [15] to display certain features. In the simplest case, we can approximate this problem through a single iteration of an alternating optimization scheme by splitting the dual optimization into two independent sub-problems. We first find the set of synthetic points $Z^{*}$ and then optimize the copy parameters $\theta^{*}$ accordingly. Figure 1 shows an example of this, where the original training data points are fed to a neural network. A synthetic dataset is generated by sampling the attribute domain uniformly at random and labeling the samples according to the predictions of the neural net. The resulting dataset is then used to build a copy decision tree that yields a performance very similar to that of the original net. Formally, the single-pass methodology can be written as shown in Algorithm 1. The algorithm follows three main steps.

- **Step 1: Synthetic sample generation**. This step of the process accounts for finding the optimal set of data points for the copy. Because we approximate this step with a single iteration of an alternating scheme, it suffices to draw samples from a distribution $P_{Z}$ that represents the coverage properties of the space where we want to build the copy with confidence (For high-dimensional synthetic samples, we can simply use $P_{Z}=N\left(0,I\right)$).
  $$Z^{*}={\left\{z_{j}\right\}}_{j=1}^{N},\;z_{j}∼P_{Z}.$$
- **Step 2: Label the synthetic set of samples.** The optimal set $Z^{*}$ is labeled according to the class prediction outputs of $f_{O}$. This defines the synthetic dataset,
  $$Z={\left\{\left(z_{j},f_{O}\left(z_{j}\right)\right)\right\}}_{j=1}^{N}$$
- **Step 3: Finding the optimized model**. Given a synthetic set Z, this step finds the value of the parameters of the copy model $\theta^{*}$ using regularized empirical risk keeping $Z^{*}$ constant, as follows:
  $$\begin{array}{cc} \theta^{*} & =\arg\min_{\theta }\left[\frac{1}{N}\sum_{j=1}^{N}\gamma_{1}\ell_{1}\left(f_{C}\left(z_{j},\theta \right),f_{O}\left(z_{j}\right)\right)+\gamma_{2}\ell_{2}\left(\theta ,\theta^{+}\right)\right] \end{array}$$

**Algorithm 1:** Simplified single-pass copy.    **Input:**$f_{O}$, $f_{C}\left(\theta \right)$    **Output:**$\theta^{*}$1Draw *N* samples: $$\;\;Z^{*}={\left\{z_{j}\right\}}_{j=1}^{N},\;z_{j}∼P_{Z}$$2Label $Z^{*}$: $$\;\;Z={\left\{\left(z_{j},f_{O}\left(z_{j}\right)\right)\right\}}_{j=1}^{N}$$3**with $(z_{i},y_{i})\in Z$:**$$\;\;\theta^{*}=\arg\min_{\theta }\frac{1}{N}\sum_{j=1}^{N}\gamma_{1}\ell_{1}\left(f_{C}\left(z_{j},\theta \right),y_{j}\right)$$

By means of this process, differential replication can be achieved. The example in Figure 1 shows one of the main characteristics of differential replication through copying. As the model and copy need not belong to the same family, the copying process can be exploited to endow the original model with new features and characteristics. In particular, one could, as above, replicate the decision behavior of a black-box classifier using a copy that is self-explanatory. Alternatively, copies can be used to evolve from batch to online learning settings [68]. This extends a model’s lifespan as it enables adaptation to data drifts or performance deviations. Equivalently, in cases where new class labels appear during a model’s deployment in the wild, copies can be used to evolve from binary to multiclass classification settings [69]. Besides, copies have also been shown to mitigate the bias learned by models based on sensitive information [70]. In what follows, we describe how machine learning copies can be applied in highly regulated environments, such as that of credit scoring, where the regulatory framework requires, among other things, that models be interpretable.

## 4. Use Case

Residential mortgages, being one of the most common types of lending [71], constitute a major source of risk for any bank; more so when loan applicants are non-clients. In such cases, there exists no previous active contract between the bank and the borrower at the time of loan application. As a result, when deciding whether to grant or deny a loan, the bank lacks any previous data records and needs to rely on data mostly declared by the applicants themselves. A situation which greatly increases the risks associated with this type of money lending.

In this article, we study non-client mortgage loan default prediction. In particular, we discuss how models designed for this purpose can be adapted to ensure compliance with the existing regulatory framework. We explore two different scenarios where such credit scoring models could be delivered. In both cases, we study the shortcomings of an initial solution from a regulatory perspective and discuss approaches to deliver interpretable machine learning solutions that yield a good prediction performance for this problem, while complying with regulatory requirements. In Scenario 1, we use copies to ensure the attributes of a preprocessed risk scoring logistic regression model remain intelligible. In Scenario 2, we avoid the preprocessing step by exploiting a more complex, high capacity model and then copying it with a simpler one whose understandability we can control. In both cases, we ensure a good predictive performance is retained during the copying process. In what follows we briefly describe both approaches.

### 4.1. Scenario 1: De-Obfuscation of the Attribute Preprocessing

In the first scenario, we assume a standard risk modeling production pipeline. We preprocess the original dataset to train a logistic regression on a reduced set of highly predictive attributes. In a real setting, in order to find a valid set of predictive variables, a qualified risk analyst would have to conduct a tedious process of trial and error. This incurs large economical costs and a delayed time-to-market delivery. The whole process can take up to six months. Even worse, this preprocessing largely reduces interpretability of the learned model, as the new variables often reflect complex relations among the original data attributes that cannot be easily explained. Indeed, while the logistic regression itself may be linear, the relations encoded by the preprocessed variables are nonlinear, to ensure that the final model can capture complex patterns in the data. As a result, the proposed solution may fail to meet the requirements imposed by the financial regulator.

**Figure 2:**
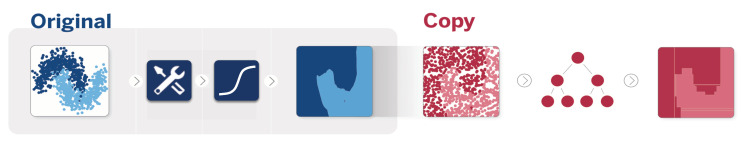
Diagram for Scenario 1, where we copy the whole predictive system, composed by both the preprocessing step and the logistic regression classifier, and obtain a new classifier applied directly on the decomposable raw data attributes.

To overcome this issue, we propose a solution based on copying the whole predictive system using a decision tree classifier, as shown in Figure 2. We substitute the original pipeline with a nonlinear model that is directly applied on the raw input features and which outputs the prediction labels learned by the original logistic model. As a result, we obtain explanations directly on the original data attributes, which are more easily understandable.

### 4.2. Scenario 2: Regulatory Compliant High Performance Copies

**Figure 3:**
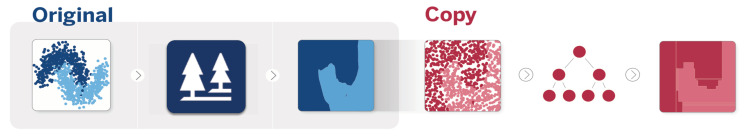
Diagram for Scenario 2, where we remove the preprocessing step and instead train a higher performance tree model on the original data attributes. We then copy this classifier by means of a self-explanatory model to extract global explanations.

In the second scenario, we propose an alternative approach to the same problem by completely avoiding the preprocessing step. We exploit a more complex model with a higher learning capacity and then copy it with a simpler algorithm that enables the resulting copy to be interpretable under certain assumptions. A diagram of this process is shown in Figure 3. We use the whole set of raw attributes to train a gradient-boosted tree. Because of the boosting step, the resulting model is not easily interpretable. Therefore, we copy it using a standard decision tree that retains most of the obtained prediction accuracy, while remaining understandable and hence complying with regulatory requirements.

## 5. Experiments

In this section, we describe our experiments for the two different use case scenarios proposed. We begin by discussing the experimental setup, including dataset preprocessing and original model training, and then move on to discuss the synthetic sample generation process in order to build copies that satisfy the identified needs.

### 5.1. Dataset

We use a private dataset of non-client loan applications recorded by BBVA during 2015 all over Mexico. At the time of loan application, all individuals in this dataset were considered by the bank to be creditworthy and therefore granted a mortgage loan. However, only 1025 of them paid it off, which corresponds to a percentage of defaulted loans of 23%. The average percentage of defaults in the Mexican mortgage market for the first, second, third, and fourth quarters during the years 2015, 2016, and 2017 was 2.7% [72].

The complete dataset consists of the 18 attributes listed in Table 1 for 1.328 non-client applicants. The data include information about loan characteristics, including the total amount and the duration, together with socio-demographic and financial information about the applicants. The attributes include both data declared by the applicants at the time of loan application and supplementary information collected by the bank. When it comes to each applicant’s income, for example, the dataset includes three different values. The *est_income* include the income declared by applicant. The *est_soc_income* refers to the income estimation made by the bank in terms of each applicant’s socio-demographic profile. Finally, the *est_mila_income* consists of the salary estimated by the Mexican Treasury Ministry for each individual in the dataset. In addition to these, *economy_level* is a categorical variable internally computed at the bank to classify clients depending on their level of income. It consists of 7 different levels. Most individuals in the dataset are assigned to the lower economy level, which corresponds to a low to average income, and only a very small amount being assigned to the highest levels.

Due to proprietary reasons, the original dataset cannot be made public. A reconstruction of this dataset that recovers the mean, standard deviation, covariance, and correlations, as well as the label distribution can be found in [4].

### 5.2. Experimental Settings

Due to the sensitive nature of bank data, we anonymize all the records using randomly generated IDs. We convert all nominal attributes to numerical and re-scale all attributes to the [0,1] range. We perform a stratified split to obtain training and test sets with relative sizes of 0.8 and 0.2, respectively, and train the original models directly on these data.

As explained before, we suggest two different approaches to deliver interpretable machine learning solutions that yield a good prediction performance for this dataset while complying with regulatory requirements. In Scenario 1, we use copies to ensure the attributes of a risk scoring model remain intelligible. In Scenario 2, we avoid the preprocessing step by exploiting a higher learning capacity model and then copying it with a simpler yet interpretable one.

In both cases, we create synthetic datasets by drawing random samples from a uniform distribution defined over the given attribute domain. We label these samples according to the predictions output by the classifiers trained on the original data. In the case of Scenario 1, we sample synthetic data points directly in the original attribute domain and label them using both the preprocessing module and the trained logistic regression model. In both scenarios, we build synthetic sets comprised of $N=1\times 10^{6}$ samples and use these data to train copies based on decision tree.

We train all models using *scikit-learn* default parameters. For the case of the logistic regression model trained in scenario 1, we use L2 penalty, a tolerance of $10^{-4}$ and the limited-memory BFGS solver. Following the discussion in Section 3, we enforce no capacity control on the copies and use misclassification error as the criterion for splitting when building the copy decision trees.

### 5.3. Validation and Discussion of Results

We evaluate copies using *copy accuracy*. This value corresponds to the accuracy of the copy in the original test data [15]. For validation purposes, we also generate an additional test set composed of $N=1\times 10^{6}$ synthetic samples and report metrics on this set. We use the performance on this second set as a safety check, to ensure that copies replicate the original models’ decision behavior even in face of previously unseen data. In all cases, we report results averaged over independent 100 runs.

In the first scenario, we manually craft the four variables shown in Table 2. These variables are based on combinations among the raw features. We use these four artificially crafted attributes together with *age* and *economy_level* to train a logistic regression model. The whole predictive system is composed of both the preprocessing step and the logistic model. The final model, trained on the reduced set of predictive attributes, yields an accuracy of 0.77 with precision of 0.68 and recall of 0.64. These results are in line with our expected outcomes, considering that our setting deals with non-client data and therefore many attributes in the dataset are not backed by objective evidence and have been instead estimated or declared by the applicants themselves. Additionally, precision and recall suggest that the original classifier does not display any pathology.

**Figure 4:**
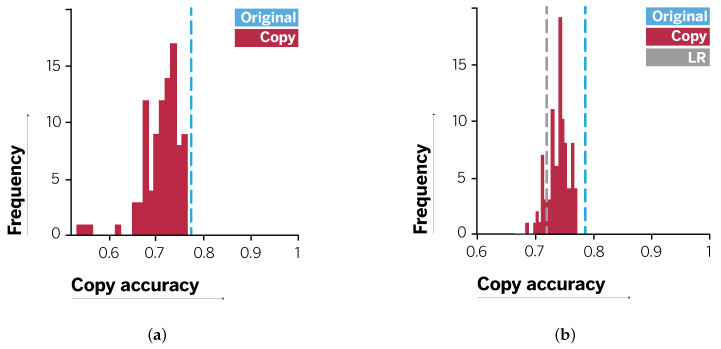
Distribution of copy accuracies for scenarios (**a**) 1 and (**b**) 2.

The distribution of copy accuracy results for the decision tree classifiers in this scenario is shown in Figure 4a. This plot shows the variance of results for copy decision trees built on different sets of synthetic data points. In all cases, metrics are measured over the original test data points. The mean copy accuracy over all runs is 0.71±0.04 with mean precision of 0.62 and recall of 0.56. This corresponds to a loss of accuracy of 0.6 points over the original logistic pipeline. This loss is also displayed in the precision and recall scores. Observe that the copy distributes the loss among both.

While this loss may seem substantial, note that the results reported here correspond to reduced synthetic datasets composed of $10^{6}$ samples. Moreover, these samples have been generated assuming a uniform probability distribution throughout the attribute domain. Using more sophisticated sampling techniques may ensure a better representation of the original decision boundary in the synthetic data and hence yield better accuracy results [73].

Note, however, that as they stand our results show that it is possible to copy the original prediction pipeline to a reasonable level of fidelity. Moreover, as a result of this process we can provide new explanations based on the original raw data attributes and thus maintain the decomposability. Further, as we will see for the next scenario, using decision tree-based copies allows us to control the accuracy–interpretability trade-off by choosing the desired tree depth when copying.

**Figure 5:**
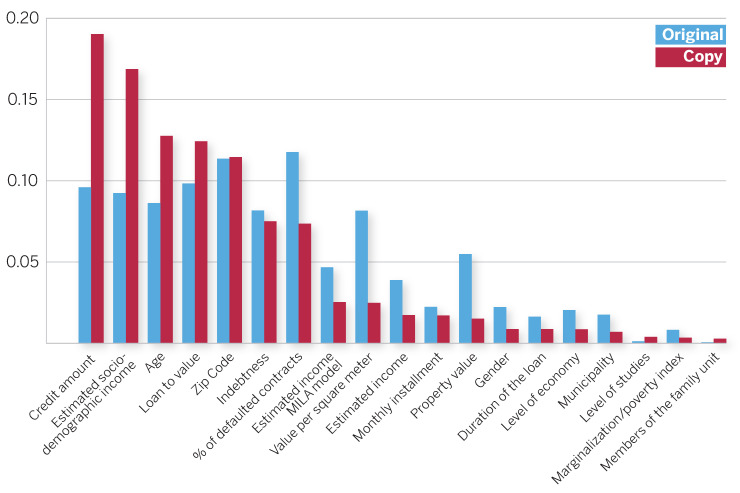
Feature importances for the original gradient-boosted tree (blue) and the copy decision tree (red).

In Scenario 2, we use all the 18 attributes listed in Table 1 to train a gradient-boosted tree. This model yields an accuracy of 0.79 with precision of 0.65 and recall of 0.61. These values are sensibly higher than those obtained by the preprocessed logistic regression in Scenario 1. This is because the learned decision function can capture nonlinear relationships among original data attributes. The copy decision trees yield a mean accuracy of 0.739±0.018 for original test data, with precision of 0.61 and recall of 0.57. Although, these values may seem low, the fidelity in recovering the original confusion matrix is high. The original confusion matrix yields the following results: TP = 185, FP = 20, FN = 42, and TN = 19. The confusion matrix for the copy decision trees averaged over all runs is given by TP = 188, FP = 17, FN = 48, and TN = 13. The corresponding distribution of copy accuracy scores is displayed in Figure 4b, where we also show the accuracy obtained by a logistic model directly on the raw attributes, which is equal to 0.72. Besides, in Figure 5 we also show the feature importances for both the original and the copy models, ranked in terms of the latter. Even while punctual differences can be observed in the importance scores of certain variables, both models agree on the most important attributes. Notably, the distribution of importance scores is more skewed for the copy, meaning that after the copying process, errors are minimized from a global perspective making it possible to better discriminate among attributes.

**Figure 6:**
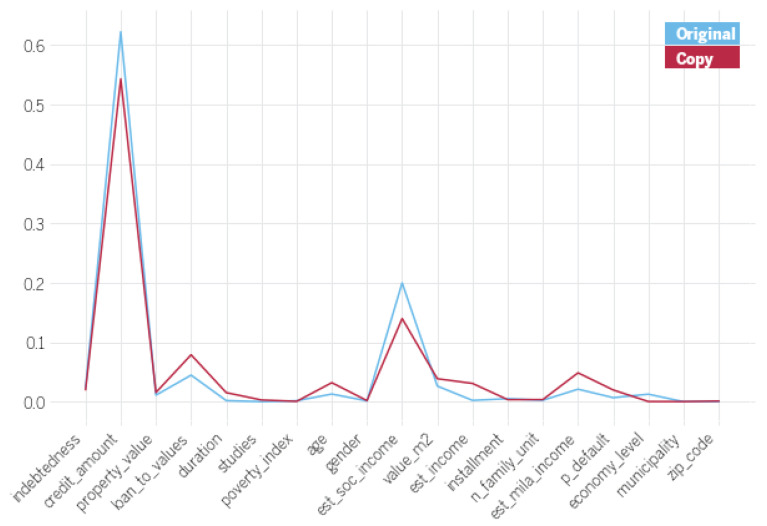
Comparison of normalized first order coefficients for original (blue) and copy models (red).

To further validate the copying results, a sensitivity analysis of this scenario is performed. Figure 6 shows the normalized first order coefficients for the different attributes in the original training set for both the original high capacity model and the decision tree-based copies. First-order sensitivity indices have been computed according to *Sobol* sensitivity analysis with 10,000 samples generated using the *Saltelli* sampler. Note that, small differences aside, coefficient values for the copy are reasonably similar to those of the original model. We attribute these differences to the inability for the copy to exactly replicate the boundary of the extreme gradient boosted tree.

Finally, we explore the interpretability-accuracy trade-off in greater detail. While decision trees are widely considered to be self-explanatory, the level of understandability of these models is highly dependent on their size. The deeper the trees, the greater the number of decision cuts, the more intricate they are. Conversely, the shallower the trees, the easier they are to understand for a non-educated audience. Therefore, we may ensure a higher level of understandability for the client by building copies based on shallow decision trees. However, this implies a process of simplification, which may come at the cost of accuracy.

In a business context, like the one at hand, it is important not only that we ensure that credit models are interpretable, but also that the risk derived from overly simplifying a given model is properly measured. In this article, we show how copies can be exploited for this purpose.

**Figure 7:**
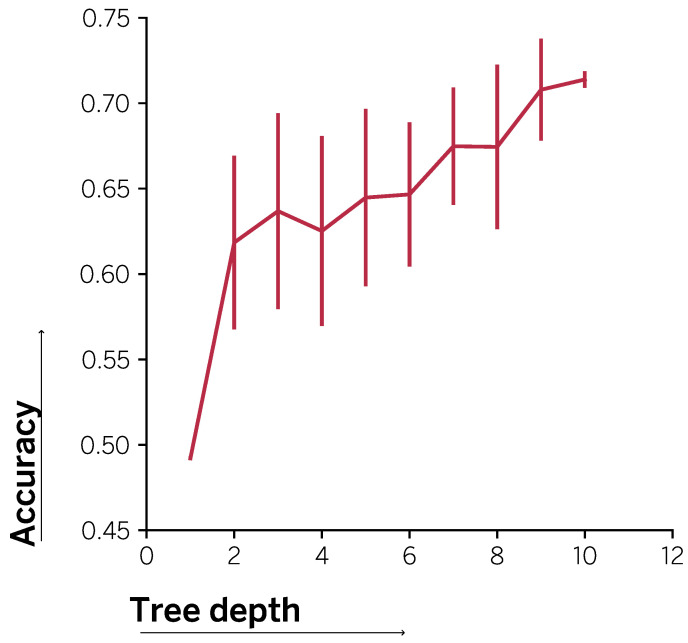
Accuracy for different copy depths.

In Figure 7, we show the accuracy yielded by copies based on decision trees of increasing depths. In all cases, we show results averaged over 10 runs. The smallest trees compact all the information in the original solution into a single layer that captures the most variability. In this use case, the decision node obtained corresponds to the *credit_amount*. This insight is in correspondence with the feature with the highest relevance in the gradient boosted tree, as shown in Figure 5. As the number of layers increases, so does the amount of information captured by the copy, which grows richer as more layers are added. Again, analyzing the most important features in the path of the trees, we observe that the next best feature is *est_soc_income*. However, as the depth of the tree increases the differences between the feature relevance for the original model and the inner decision features selected by the copy decision trees are more apparent. In this regard, note that the different copy trees are trained using different synthetic sets. Differences in the samples included in each set can therefore lead to varying decision paths for the trees. In this sense, the greater the depth, the higher the variability of the splitting features from tree to tree.

Nevertheless, the deeper the trees, the better they perform. Note that fully grown trees obtain an average accuracy of 0.739±0.018 as mentioned before. Equivalently, the more understandable the trees, the smaller the depth, the higher the loss in accuracy. Therefore, Figure 7 shows how copies can be used to provide explanations of varying levels of complexity, while at the same time controlling the associated accuracy loss. These can be used to provide appropriate explanations to clients in cases where they demand so. However, also, to provide data scientists or computer engineers with understandable copies to aid in the process of monitoring a given solution.

## 6. Conclusions

In this paper, we show how differential replication through model-agnostic copies can be used to tackle the problem of interpretability in credit risk scoring. Copies endow models with new features not present in original models while retaining their performance. We briefly sketch the particularities of the optimization process for building a copy and discuss the reasons why copying departs from standard machine learning assumptions and practices. Further, we describe how differential replication through copies can be used in the context of credit scoring models and propose a real use case for non-client mortgage loan default prediction. Finally, we validate our proposal in two scenarios: deobfuscation of the feature domain engineering and regulatory-compliant high-performance copy building. We show that differential replication can be exploited to effectively bridge the trade-off between accuracy and interpretability by generating copies that display both.

We conclude this paper by identifying open challenges that deserve further attention. There exist two areas where differential replication through copies could be applied: For one, in settings where models need to be made available to the public while preserving the privacy of the original training data, copies could be used to obfuscate the individual data points. Besides, we would also like to study the applicability of copies for causal inference, to build actionable counterfactual frameworks.

## Figures and Tables

**Table 1:** Complete set of attributes.

Attribute	Description
*indebtedness*	Level of indebtedness
*credit_amount*	Amount of credit
*property_value*	Property value
*loan_to_value*	Loan to value
*duration*	Duration of the loan
*studies*	Level of studies
*poverty_index*	Marginalization/poverty index
*age*	Age
*est_soc_income*	Estimated socio-demographic income
*value_m2*	Value per square meter
*est_income*	Estimated income
*installment*	Monthly installment
*n_family_unit*	Members of the family unit
*est_mila_income*	Estimated income based on MILA model
*p_default*	Percentage of defaulted contracts in the last 4 months from those signed during the previous 12 to 24 months
*zip_code*	ZIP code
*municipality*	Municipality
*economy_level*	Level of economy

**Table 2:** Reduced set of highly predictive attributes.

Attribute	Description
*zip_code_municipality*	Bivariate attribute resulting from the concatenation of features zip_code and municipality
*est_soc_income/est_mila_income*	Univariate attribute resulting from the ratio between features est_soc_income and est_mila_income
*property_value/installment*	Univariate attribute resulting from the ratio between features property_value and installment
*indebtedness/loan_to_value*	Univariate attribute resulting from the ratio between features indebtedness and loan_to_value

## Data Availability

The data used in this article is available upon request from the authors.
